# Mr. Graham-Bennett

**Published:** 1892-12-10

**Authors:** H. Howgrave Graham

**Affiliations:** Secretary, Hospital for Epilepsy and Paralysis, 32, Portland Terrace, Regent's Park


### MR. GRAHAM-BENNETT.

Referring to Mr. Graham-Bennett's letter respecting the "Hospital for Curing the Poor in their own Homes of Loss of Movement from Paralysis Rheumatism and Deformity," will you allow me space, as Mr. Graham-Bennett's name is rather like that of a member of our staff—Dr. Hughes-Bennett—to say that the "Paralytic Hospital," to which Mr. Graham-Bennett refers is not this institution, with which Mr. Graham-Bennett has never been connected.

H. Howgrave Graham, Secretary,
Hospital for Epilepsy and Paralysis,
32, Portland Terrace, Regent's Park.

